# The Success Rate of Endotracheal Intubation in the Emergency Department of Tertiary Care Hospital in Ethiopia, One-Year Retrospective Study

**DOI:** 10.1155/2021/9590859

**Published:** 2021-03-19

**Authors:** Ayalew Zewdie, Dejene Tagesse, Selam Alemayehu, Tesfaye Getachew, Menbeu Sultan

**Affiliations:** ^1^Department of Emergency Medicine and Critical Care, St. Paul's Hospital Millennium Medical College, Addis Ababa, Ethiopia; ^2^Addis Ababa Burn Emergency and Trauma (AaBET) Hospital, Addis Ababa, Ethiopia; ^3^Department of Psychiatry, St. Paul's Hospital Millennium Medical College, Addis Ababa, Ethiopia

## Abstract

**Background:**

Emergency medical care starts with airway assessment and intervention management. Endotracheal intubation is the definitive airway management in the emergency department (ED) for patients requiring a definitive airway. Successful first pass is recommended as the main objective of emergency intubation. There exists no published research regarding the success rates or complications that occur within Ethiopian hospitals emergency department intubation practice.

**Objective:**

This study aimed to assess the success rate of emergency intubations in a tertiary hospital, Addis Ababa, Ethiopia. *Methodology*. This was a single institute retrospective documentation review on intubated patients from November 2017 to November 2018 in the emergency department of Addis Ababa Burn Emergency and Trauma Hospital. All intubations during the study period were included. Data were collected by trained data collectors from an intubation documentation sheet.

**Result:**

Of 15,933 patients seen in the department, 256 (1.6%) patients were intubated. Of these, 194 (74.9%) were male, 123 (47.5%) sustained trauma, 65 (25.1%) were medical cases, and 13(5%) had poisoning. The primary indications for intubation were for airway protection (160 (61.8%)), followed by respiratory failure (72(27.8%)). One hundred and twenty-nine (49.8%) had sedative-only intubation, 110 (42.5%) had rapid sequence intubation, and 16 (6.2%) had intubation without medication. The first-pass success rate in this sample was 70.3% (180/256), second-pass 21.4% (55/256), and third-pass 7.4% (19/256), while the overall success rate was 99.2% (254/256). Hypoxia was the most common complication.

**Conclusion:**

The intubation first-pass success rate was lower than existing studies, but the overall intubation success rate was satisfactory.

## 1. Background

Emergency medical care starts with airway assessment and intervention management. Endotracheal intubation is the definitive airway management in the emergency department (ED) for patients requiring a definitive airway. Anesthetists and or emergency physicians (EPs) perfume intubations with or without drugs [1]. Advanced airway management is a basic skill that every emergency physician (EP) should acquire worldwide [2].

The most widely used emergency airway management is rapid sequence administration of sedation and paralytic agent with tracheal intubation (rapid sequence intubation (RSI)) [3]. RSI performed in critically ill and injured patients in the emergency room has a high risk of failed intubation and other complications than that in the operating room [4, 5]. This is caused by several factors such as patients having significant comorbidities; they have not fasted and need to immobilize the cervical spine. In addition, clinical condition of the patient, the experience and training of the clinician, the urgency of the procedure, and the availability of equipment and personnel to support the procedure in the emergency room affect the success and complication of emergency intubation [2, 3].

Audit of critical emergency procedures helps to improve training, policy, and clinical practice development. In North America, surveillance studies demonstrated that ED intubations by emergency medicine residents and physicians, largely using a rapid sequence intubation technique, can be performed safely and with high levels of success [6].

A successful first pass is recommended as the main objective of emergency intubation. Study from the anesthesia literature showed that repeated attempt of intubations leads to a higher complication rate [7]. There are limited data from emergency medicine in the developing world about the success of airway management in the emergency room [8]. There is a need to fill this knowledge gap in order to identify the frequency of failed intubation and its causation so that clinical outcomes can be improved.

The aim of this study was to assess the success rate of emergency intubations in the tertiary hospital, Addis Ababa, Ethiopia.

## 2. Methodology

This was a single institute retrospective documentation review on intubated patients from November 2017 until November 2018 in the ED of Addis Ababa Burn Emergency and Trauma Hospital (AaBET Hospital), a major trauma centre in Addis Ababa, which is one of the biggest teaching hospitals in Ethiopia. AaBET currently provides health care services in several specialties: orthopaedics, neurosurgery, plastic and reconstructive surgery, emergency and critical care, and general surgery. AaBET hospital had approximately 20–30,000 annual emergency room visits. It provides outpatient services as well as elective and emergency surgeries.

The emergency medicine and critical care residency program, which is a 3-year program, was started in 2015. Residents were given an induction course on emergency medicine and critical care basic topics and practical skills before they start the clinical activity. Likewise, they underwent an airway course and had practical training on manikins. The study included intubations performed with or without the use of drugs. Patients who came intubated from other hospitals were excluded.

### 2.1. Operational Definitions

“An episode of intubation was defined as the process of intubation for each patient, whereas an attempt at intubation was defined as a single passage of the laryngoscope blade past the lips” [2].

“Difficult laryngoscopy was defined as Cormack and Lehane grade III or IV [2] and difficult intubation as one that requires more than two attempts. “Complications of orotracheal intubations were defined as peripheral oxygen saturation (SpO_2_) <93%, even if the patient was hypoxic before intubation and had bradycardia<60/min, hypotension requiring support, airway trauma, esophageal or main stem bronchial intubation, laryngospasm, cardiac arrest, or the performance of a surgical airway” [2].

### 2.2. Data Collection

All intubations in the ED were documented by ED nurse using a purpose designed documentation sheet (Annex-1) as close to the time of intubation as possible. The provider who intubated the patient signed the form after data were filled by the nurse. The documentation form was used by the staff during intubation as a standard protocol of the hospital.

We developed another form (Annex-2) for our analysis which includes age, sex, diagnosis, indication for intubation, anesthetic agents used for intubation, pre- and postintubation vital signs (V/s), immediate complications, and the number of intubation attempts, the intubation personnel, and outcome of intubation.

### 2.3. Statistical Analysis

Intubation data between November 2017 and November 2018 for one year were analyzed using SPSS version 21.

Descriptive statistics were performed on months of age, sex, diagnosis on admission, indication for intubation, number of patients in whom intubation was attempted, and number of attempts. The study was reviewed and approved by the hospital, Emergency Medicine Department Institutional Review Board.

## 3. Results

**Figure 1:**
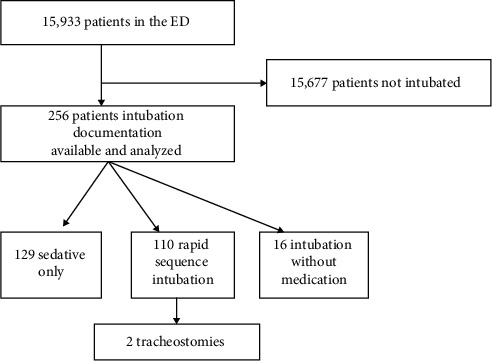
Flow chart showing data analysis of emergency intubations.

Out of 15,933 patients, a total of 256 intubations (1.6%) were performed in the study period (Figure 1). One hundred ninety-four (75.8%) of the intubated patients were male, and 62 (23.9%) were female. The median age was 30 years with an interquartile range of 20 and 49 (Table 1).

### 3.1. Diagnosis and Indication for Intubation

**Figure 2:**
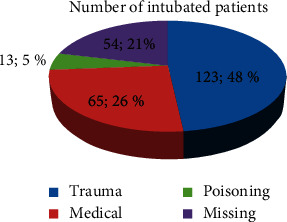
Indications of intubated patients of AaBET hospital Addis Ababa, Ethiopia.

One hundred twenty-three patients (48.0%) sustained trauma (Figure 2).

The primary indications for intubation were for airway protection 160 (62.5%), followed by respiratory failure 72 (28.1%). An indication for the remainder was not documented.

### 3.2. Type of Intubation

The types of intubation used were as follows: 129 (50.3%) sedative-only intubation, 110 (42.9%) rapid sequence intubation, 16 (6.3%) intubation without medication. The most common induction agent used was ketamine, and the only paralytic agent was succinylcholine (Table 2).

**Figure 3:**
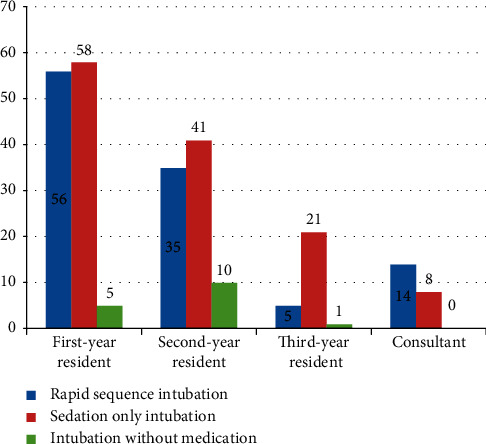
Level of intubators and type of intubations at AaBET hospital, Addis Ababa, Ethiopia.

First-year residents used RSI in 56 (50.9%) patients, sedation only intubation in 58 (45.0%), and intubation without medication in 5 (31.3%) patients during first attempt of intubation (Figure 3).

### 3.3. Attempt and Success Rate

The first-pass success rate in this sample was 70.3% (180/256), second-pass 21.4% (55/256), and third-pass 7.4% [9], while the overall success rate was 99.2% (254/256). There were only two failed intubations in the one-year period after trial of RSI that required emergency tracheostomy for the diagnosis of tracheal stenosis, and they were performed by surgeons.

From 254 successful intubations, 122 (48.0%), 66 (25.9%), 34 (13.3%), and 32 (12.5%) were intubated by second-year residents, first-year residents, consultants, and third-year residents, respectively (Table 3).

### 3.4. Complications of Intubation

The overall complication rate was 28.5% (73/256). Thirty-two patients had hypoxia with SpO_2_ < 93%, and 25 had hypotension (Table 4).

Type of intubation versus demographics, level of intubators, final success of intubation, and complication is shown in Table 5.

## 4. Discussion

This was the first descriptive study on intubation in the emergency department of AaBET hospital.

In the finding of this study, all ED intubations were managed by emergency residents and physicians. This was not the case in other countries [2, 10].

Our study demonstrated poor success rates for first-year residents on the initial attempt related to the other senior residents. On the contrary, the success rate of 70.3 % on the first attempt was not satisfactory and lower than that of studies from other countries, in which the success rate ranged from 80% to 90% [2, 11–14]. Besides, the majority of the intubated patients had trauma, which could increase the difficulty in intubation and necessitates further training and practice. Lastly, all intubations were done by direct laryngoscope because there was no video laryngoscope in the hospital which could have improved first-pass success [15]. The first-attempt success rate was higher than reports from Japan on intubated trauma patients, where the overall first attempt success rate was 63.8% [16].

The most common indications for intubation were airway protection (61.8 %), followed by respiratory failure. This could be because most of the intubated patients were trauma patients (123/256).

Also of note, intubation with sedation (129/256) was somewhat more frequent higher than rapid sequence intubation (110/256) in this study. Most studies recommend RSI in the emergency room instead [6, 17, 18]. In our opinion, this may be because most patients had trauma requiring airway protection anticipating possible difficulty in intubation or because of fear of paralytic drug complication likely implying a lack of confidence in the ability to deal with prolonged apnea. In this study, even though first-year residents used RSI for intubation more frequently than senior residents, the first-year success rate was poor, which suggests, as would be expected, that experience is more important than the type of intubation. However, further study is needed for why intubation with sedation is used more by intubators. Studies showed first attempt success was higher with RSI [9, 19, 20]. Based on these studies, the hospital intubators require quality improvement projects and should follow standard recommendations during intubation.

The most frequent agent used as a sedative was ketamine. Other studies have used mostly etomidate as a sedative [1], which was not totally available in our setting. Nonetheless, studies had shown ketamine as a safe alternative to etomidate with no side effect of adrenal suppression [21].

The most commonly used paralytics were succinylcholine, as in other studies [7, 22, 23].

Oxygen desaturation was the most common complication, similar to other studies. This study showed that multiple attempts were associated with complications like other studies [7]. Studies suggested proper preoxygenation and apneic oxygenation with nasal cannula will improve desaturation and also first-pass success rate [24–26].

Above all, the hospital should have intubation protocol on type of intubation, rescue devices, and others to improve the success rate and to decrease intubation-related complications.

## 5. Limitations

There were several limitations to this study. First and foremost, there was incomplete data for analysis and therefore underlying causes of incomplete data should be explored, with improvements made as needed.

The second limitation was the authors reviewed only the documented intubation sheets, and it was difficult to confirm if all intubations were documented. This could lead to a possible underestimation of overall intubations and other events.

Lastly, the study was not designed to assess the outcome of the intubated patient. Future studies are needed with follow-up of patients to assess mortality and morbidity.

## 6. Conclusions

The intubation first-pass success rate of AaBET Hospital was lower than that of existing studies, but the overall intubation success rate was satisfactory.

## Figures and Tables

**Table 1:** Demographics and frequency of intubations at Addis Ababa, Ethiopia.

Variables	Subclass	Frequency and percentage (*N* = 256), *n* (%)
Age	<18	37 (14.5)
	≥18	196 (76.5)
	Missing	23 (9.0)
Sex	Male	194 (75.8)
	Female	62 (24.2)
Preintubation GCS	3–8	111 (43.4)
	9–12	25 (9.8)
	13–15	43 (16.8)
	Missing	80 (30.0)
Preintubation SBP	<90	17 (6.6)
	90–120	73 (28.5)
	120–160	119 (46.5)
	>160	27 (10.5)
	Missing	23 (8.9)
Preintubation PR	<60	23 (8.9)
	60–100	89 (34.8)
	101–150	110 (43.0)
	>150	17 (6.6)
	Missing	17 (6.6)
Preintubation RR	<12	1 (0.4)
	12–24	69 (26.9)
	24–35	56 (21.8)
	>35	44 (17.1)
	Missing	86 (33.8)
Preintubation SpO_2_	<90	73 (28.5)
	≥90	172 (67.2)
	Missing	11 (4.3)

*Index.* PR, pulse rate; SBP, systolic blood pressure; RR, respiratory rate; GCS, Glasgow Coma Scale.

**Table 2:** Medications used during intubations at Addis Ababa, Ethiopia.

Medication class	Name of medication	RSI, *n* = 110(%)	SED, *n* = 129(%)	Frequency (*N* = 256) (%)
Induction/sedative	Ketamine	98(89.1)	84(65.1)	182(71.1)
	Ketamine with diazepam	3(2.7)	22(17.1)	25(9.8)
	Propofol	4(3.6)	14(10.8)	18(7.0)
	Thiopental	4(3.6)	4(3.1)	8(3.1)
	Ketamine with propofol	1(0.9)	5(3.9)	6(2.3)
Paralytics	Succinylcholine	110	0	110(42.9)

**Table 3:** First attempt and success rate of intubations at Addis Ababa, Ethiopia.

Intubator	Number of first attempt (*N* = 256), *n* (%)	Successful first attempt from first attempt, *n* (%)	Final number of successful intubations (*N* = 256), *n* (%)
First-year resident	120 (46.9)	64/120 (53.3)	66/256 (25.8)
Second-year resident	87 (34.0)	75/87 (86.2)	122/256 (47.7)
Third-year resident	27 (10.5)	22/27 (81.5)	32 (12.5)
Consultant	22 (8.6)	19/22 (86.4)	34 (13.3)
Total	256 (100)	180/256 (70.3)	254 (99.2)

**Table 4:** Immediate complications of intubations at Addis Ababa, Ethiopia.

Complications	RSI (*n* = 110), *n* (%)	Non-RSI (*n* = 146), *n* (%)	Total (%) (*N* = 256)
(SpO_2_) **<**93%,	13 (11.8)	19 (13.0)	32 (12.5)
Hypotension	6 (5.5)	19 (13.0)	25 (9.8)
Bradycardia**<**60/min	3 (2.7)	9 (6.2)	12 (4.7)
Esophageal intubation	5 (4.5)	2 (1.4)	7 (2.7)
Difficult intubation	4 (3.6)	1 (0.7)	5 (1.9)
Laryngeal spasm	1 (0.9)	1 (0.7)	2 (0.8)
Surgical airway(tracheostomy)	0 (0)	2 (1.4)	2 (0.8)
Self-extubation	1 (0.9)	0 (0)	1 (0.4)
Vomit appearing in the airway after induction	Not documented	Not documented	Not documented

**Table 5:** Type of intubations versus different variables at Addis Ababa, Ethiopia.

Variables		Medication use/type of intubation		
		RSI (*N* = 110), *n* (%)	SED (*N* = 129), *n* (%)	IOM (*N* = 16), *n* (%)
Age	<16	13 (11.8)	16 (12.4)	2 (12.5)
	17–35	45 (49.9)	50 (38.8)	8 (50.0)
	36–65	37 (33.6)	35 (27.1)	5 (31.3)
	>65	6 (5.5)	13 (10.1)	1 (6.3)
Sex	Male	79 (71.8)	100 (77.5)	12 (75.0)
	Female	30 (27.3)	27 (20.9)	4 (25.0)
	Not documented	1 (0.9)	2 (1.6)	0 (0)
First attempt	First-year resident	56 (50.9)	58 (45.0)	5 (31.3)
	Second-year resident	35 (31.8)	41 (31.8)	10 (62.5)
	Third-year resident	5 (4.5)	21 (16.3)	1 (6.3)
	Consultant	14 (12.7)	8 (6.2)	0 (0)
First attempt success	Successful	75 (68.2)	90 (69.8)	14 (87.5)
	Not successful	34 (30.9)	38 (29.5)	2 (12.5)
Final intubation	First-year resident	34 (30.9)	30 (23.3)	3 (18.8)
	Second-year resident	46 (41.8)	58 (45.0)	12 (75.0)
	Third-year resident	9 (8.2)	24 (16.6)	1 (6.3)
	Consultant	19 (17.3)	13 (10.1)	0 (0)
	Not documented	2 (1.8)	4 (3.1)	0 (0)
Final intubation success	Successful	108 (98.2)	128 (99.2)	16 (100)
	Not successful	2 (1.8)	0 (0)	0 (0)
Complication	Yes	36 (32.7)	50 (38.6)	10 (62.5)
	No	74 (67.3)	79 (61.2)	6 (37.5)

*Index.* RSI, rapid sequence intubation; SED, intubation with only sedation; IOM, intubation without medication.

## Data Availability

The data used to support the findings of this study are available upon request to the corresponding author.

## Abbreviations

AaBET Hospital: Addis Ababa Burn Emergency and Trauma Hospital
AEs: Adverse events
ED: Emergency department
EM: Emergency medicine
EPs: Emergency physicians
GCS: Glasgow Coma scale
PR: Pulse rate
SBP: Systolic blood pressure
RSI: Rapid sequence intubation
RR: Respiratory rate
V/s: Vital sign.
